# Using computer vision on herbarium specimen images to discriminate among closely related horsetails (*Equisetum*)

**DOI:** 10.1002/aps3.11372

**Published:** 2020-07-01

**Authors:** Kathleen M. Pryer, Carlo Tomasi, Xiaohan Wang, Emily K. Meineke, Michael D. Windham

**Affiliations:** ^1^ Department of Biology Duke University Durham North Carolina 27708 USA; ^2^ Department of Computer Science Duke University Durham North Carolina 27708 USA; ^3^ Department of Entomology and Nematology University of California Davis California 95616 USA

**Keywords:** deep learning, digitized herbarium specimens, Equisetales, ferns, horsetails, machine learning

## Abstract

**Premise:**

*Equisetum* is a distinctive vascular plant genus with 15 extant species worldwide. Species identification is complicated by morphological plasticity and frequent hybridization events, leading to a disproportionately high number of misidentified specimens. These may be correctly identified by applying appropriate computer vision tools.

**Methods:**

We hypothesize that aerial stem nodes can provide enough information to distinguish among *Equisetum hyemale*, *E. laevigatum*, and *E* *. ×ferrissii*, the latter being a hybrid between the other two. An object detector was trained to find nodes on a given image and to distinguish *E. hyemale* nodes from those of *E. laevigatum*. A classifier then took statistics from the detection results and classified the given image into one of the three taxa. Both detector and classifier were trained and tested on expert manually annotated images.

**Results:**

In our exploratory test set of 30 images, our detector/classifier combination identified all 10 *E. laevigatum* images correctly, as well as nine out of 10 *E. hyemale* images, and eight out of 10 *E. ×ferrissii* images, for a 90% classification accuracy.

**Discussion:**

Our results support the notion that computer vision may help with the identification of herbarium specimens once enough manual annotations become available.

The role of herbaria in scientific research has expanded far beyond what could ever have been imagined at their initiation over four centuries ago (Heberling and Isaac, 2017; Meineke et al., 2018a, b). For example, the online availability of millions of digitized herbarium specimen images and data through iDigBio (http://www.idigbio.org) has greatly facilitated novel approaches to phenological and ecological research (Soltis, 2017). Recent studies also show much promise in the application of deep learning techniques on digitized herbarium specimens for automated species identification (Schuettpelz et al., 2017), for distinguishing between mercury‐stained and unstained specimens (Collins et al., 2018), and even for the transfer of learning across domains, e.g., training a plant species classifier on herbarium images and then applying it to images taken in the field (Carranza‐Rojas et al., 2017; Wäldchen and Mäder, 2018). Deep learning methods have raised expectations for addressing a wide array of biological questions (Christen et al., 2019). In view of these recent advances in computer vision and deep learning, and given that hundreds of thousands of high‐resolution images of fern herbarium specimens are already available online (e.g., from the SouthEast Regional Network of Expertise and Collections [SERNEC], http://sernecportal.org/portal/), we decided to investigate whether applying an automated approach to identify specimens of *Equisetum* L. might benefit research related to this lineage. Due to significant intraspecific variation of visually diagnostic traits in *Equisetum* (Hauke, 1963, 1978, 1983), species‐level identifications can be challenging, leading to high numbers of misidentifications in herbaria (Moran, 1983). Because of this, we hypothesized that accurate species classification via an existing computer vision pipeline was unlikely to be successful. Here we develop a novel pipeline that provides specific guidance on what is taxonomically relevant to the classification at hand.

*Equisetum*, with only 15 extant species, is among the most phenotypically distinct vascular plant genera and has a fossil record extending back as far as the Triassic (Elgorriaga et al., 2018; Clark et al., 2019). A Cretaceous origin has been shown for the *Equisetum* crown‐group, with most extant species having originated during the Paleogene (Des Marais et al., 2003; Clark et al., 2019). Closely related species of *Equisetum* can be difficult to differentiate (Hauke 1963, 1978, 1983) because many obvious characters show a high degree of morphological plasticity, whereas diagnostic features are usually cryptic. The taxonomic situation is further complicated by frequent hybridization among closely related species. *Equisetum* hybrids are proficient at vegetative dispersal of rhizome segments, allowing these more‐or‐less intermediate forms to become broadly established geographically, sometimes beyond the range of either parental lineage. Combined, these factors have resulted in frequent species misidentifications within herbarium collections, as well as in the literature based upon them.

One of the more problematic groups of *Equisetum* involves two relatively distinct species (*E. hyemale* L. and *E. laevigatum* A. Braun) and a widespread, sexually sterile taxon (*E. ×ferrissii* Clute) shown to have arisen through hybridization between them (Rutz and Farrar, 1984; Des Marais et al., 2003). *Equisetum hyemale* is found throughout much of Eurasia (subsp. *hyemale*), as well as North America, Mexico, and Guatemala (subsp. *affine* (Engelm.) Calder & Roy L. Taylor). In terms of macroscopic distinguishing features, the nodal leaf sheaths of *E. hyemale* (Fig. 1A) are more or less cylindrical, with the rim barely wider than the internode above. Nearly all mature leaf sheaths in *E. hyemale* are prominently cross‐banded, with a dark horizontal stripe near the base, a tan to ashy white region above this, and another dark band at the rim formed by the blackish bases of the leaf teeth (the teeth themselves may be persistent or deciduous). The spore‐producing structures (strobili) at the stem apices provide additional features useful for identification; in *E. hyemale*, the strobili are apiculate (producing a sharp point distally) and dehisce when mature to release an abundance of well‐formed, greenish spores (Hauke, 1963).

**Figure 1:**
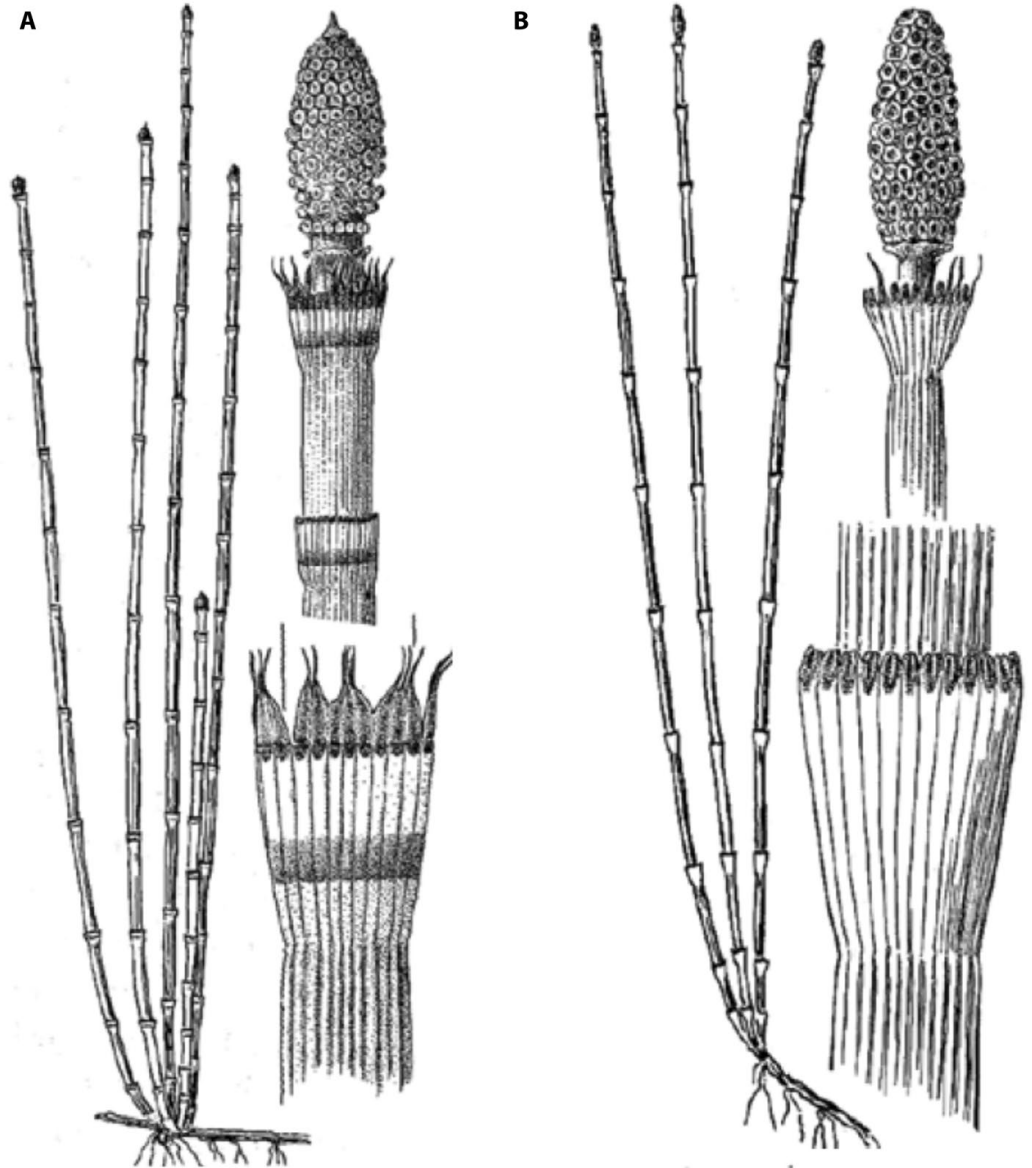
Drawings of the two sexually reproducing species of *Equisetum* included in this study. (A) *Equisetum hyemale*. (B) *Equisetum laevigatum*. Modified with permission from Hitchcock et al. (1969).

The other fertile species investigated here is *E. laevigatum*, which is broadly distributed across Canada and the United States, and extends south to northern Mexico. In *E. laevigatum* (Fig. 1B), the nodal leaf sheaths are slightly flared distally such that the rim is usually wider than the internode above. In terms of coloration, the leaf sheaths are mostly green with a narrow black rim left by the abscission of the leaf teeth. Finally, the strobili of *E. laevigatum* are non‐apiculate, although dehiscent at maturity with spores similar to those of *E. hyemale* (Hauke, 1963).

*Equisetum ×ferrissii* is a common and widespread, sexually sterile hybrid between *E. hyemale* and *E. laevigatum* that is scattered across southern Canada, the United States, and northern Mexico (Hauke, 1963). It has been reported from 38 of the 48 contiguous United States and is recorded from eight states in the Southeast and Northeast where one of its parents, *E. laevigatum*, is apparently absent (Kartesz, 2015). This hybrid is generally intermediate in morphology between its parental species but is highly variable, possibly due to multiple independent hybrid origins (Des Marais et al., 2003). The proximal nodal leaf sheaths of *E. ×ferrissii* are often banded like those of *E. hyemale* (although often irregularly so), whereas the distalmost leaf sheaths are more similar to those of *E. laevigatum*. Like its *E. hyemale* parent, *E. ×ferrissii* has apiculate strobili, but the apical point is usually less prominent. The most useful character for distinguishing *E. ×ferrissii* from its parents has been the strobili that fail to dehisce at maturity and, when broken open, contain nothing but powdery, whitish, malformed spores (Hauke, 1963).

Here we explore whether the recent confluence of mass digitization of herbarium specimens, together with rapid advances in deep learning approaches, might provide a novel automated approach to accurately identify specimens of the iconic fern lineage *Equisetum*.

## METHODS

### Annotation

**Figure 2:**
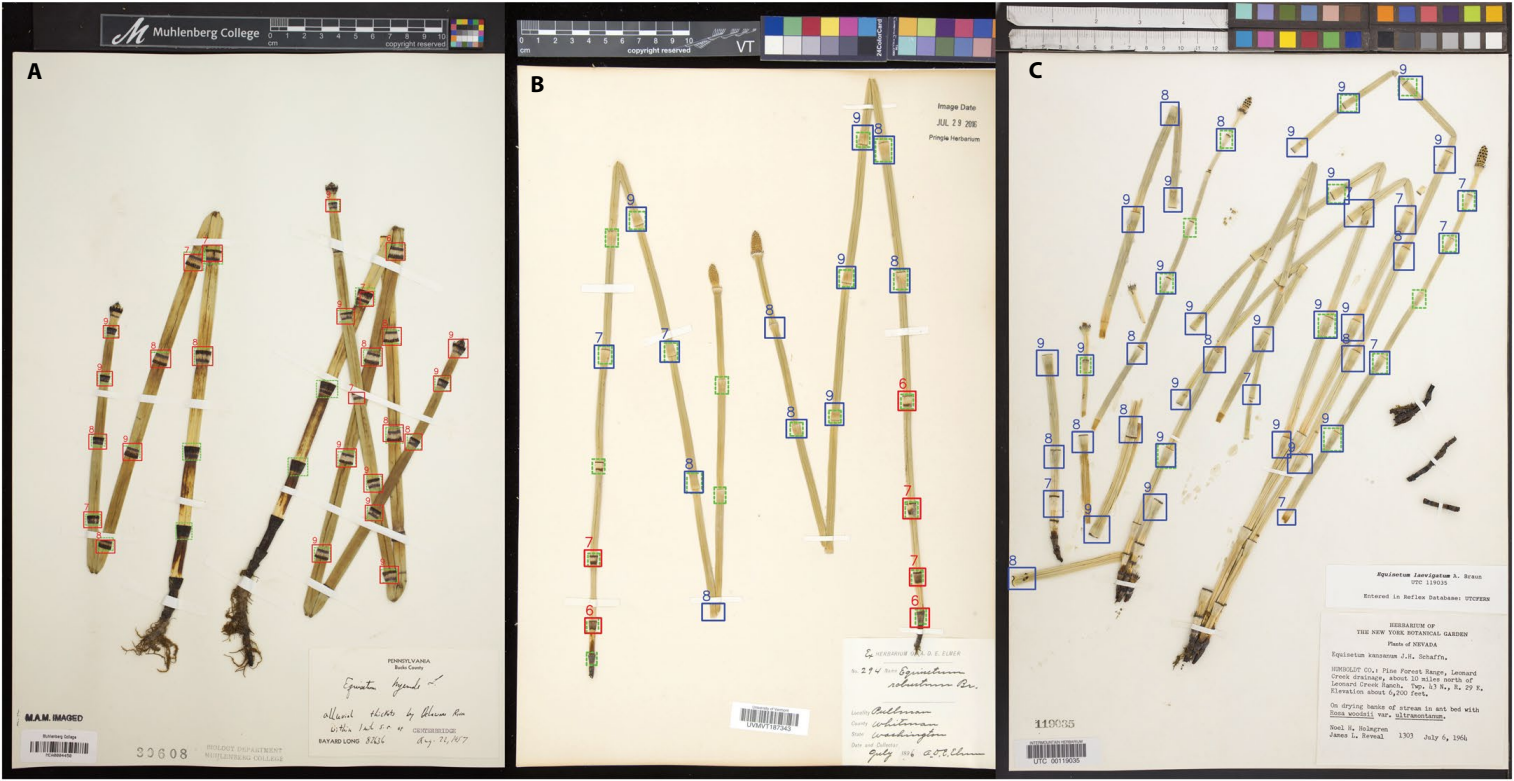
Examples of human versus machine‐applied annotations on digitized images of *Equisetum*. (A) *Equisetum hyemale* (http://sernecportal.org/portal/collections/individual/index.php?occid=13382583). (B) *Equisetum ×ferrissii* (http://sernecportal.org/portal/collections/individual/index.php?occid=11039870). (C) *Equisetum laevigatum* (http://sernecportal.org/portal/collections/individual/index.php?occid=207187). Green dashed boxes are human annotations. Solid boxes are detection results: a red box denotes a hyemale‐type (H) node; a blue box denotes a laevigatum‐type (L) node. (A) The detector missed four nodes and classified all the others correctly as being of the H variety, with high confidence scores of 6, or higher. (B) The detector missed five of the 21 nodes found by the human annotator and detected one spurious node (on the bottom kink of the stem on the left). It also found a node that had not been annotated (close to the strobilus on the stem on the right), but visual inspection shows that this is a genuine node that had not been flagged by the human annotator. Of the 18 nodes detected (genuine or spurious), the classifier determined six to be H nodes (red) and 12 to be L nodes (blue), all with high confidence scores (6, or higher). (C) Although the human annotator marked only 13 stem nodes, the classifier found many more, and classified all of them as L nodes, with high confidence scores (7, or higher). Visual inspection shows that these additional detections, which would be denoted as “false positives” in a standard evaluation (because they do not match human annotations), are all genuine nodes.

We downloaded high‐resolution, digitized specimens available for *Equisetum* from SERNEC (Appendix S1). None of the specimen identifications were initially accepted as reliable; each was critically evaluated and identified by the author (M.D.W.) with the most extensive experience identifying *Equisetum*. Thirty‐six verified specimen images were compiled for each of the three focal taxa (*E. hyemale*, *E. laevigatum*, and *E. ×ferrissii*) for a total of 108 images. We used the VGG Image Annotator version 2.0.4 (Dutta and Zissermann, 2019) to manually draw bounding boxes on the images and assign a morphological structural category to each box, out of the following four options: “strobilus,” “normal stem node,” “normal stem internode,” and “injured stem node.” Table 1 shows the number of instances annotated for each of these four categories. Annotation boxes are rectangles of arbitrary sizes that are drawn to enclose the phenotypic structure as tightly as possible. For examples of annotated images, see Fig. 2.

**Table 1:** The number of digitized images studied for each *Equisetum* species (see Appendix S1), and the number of instances that were manually annotated for each of the four structural categories investigated.

Species	No. of images studied	Strobilus	Normal stem node	Normal stem internode	Injured stem node
*E. hyemale*	36	77	791	178	2
*E. laevigatum*	36	83	479	95	33
*E*. ×*ferrissii*	36	81	827	107	0

### General approach

A typical application of currently available image classification methods for distinguishing among specimen images would build a single deep neural network that takes an image as input and then outputs one of three labels (*E. hyemale*, *E. laevigatum*, or *E. ×ferrissii*), and would then train this network on a wealth of manually annotated images. However, this approach would not work unless tens or even hundreds of thousands of manually annotated images were available (Cho et al., 2016). This is because images *within* a given species differ dramatically because of the geometry of the pressed plant stems, the number of stems included in a specimen, their colors, imaging parameters, and so forth. At the same time, what distinguishes specimens of one species from those of another has little to do with any of these macroscopic variations, but rather with subtle nuances in the appearance of stem nodes and strobili. In machine learning parlance, one would say that there is large intraclass variation and only small interclass differences. Without any specific guidance on what is relevant to the classification at hand, no classifier, deep or otherwise, is likely to do well under these circumstances.

In addition to features of the strobili, human experts who identify specimens of *Equisetum* based on macromorphology do so, by‐and‐large, by analyzing variation in nodal leaf sheaths along the length of the aerial stems. Because *E. ×ferrissii* is a hybrid, it tends to combine features of the two parental species (Fig. 1), resulting in a relatively dependable pattern of variation in node morphology. Although nodal leaf sheaths near the middle of the aerial stems in *E*. *×ferrissii* can sometimes appear intermediate between those of the parental taxa, nodes toward the base of the stem mostly resemble those of *E. hyemale* and nodes toward the apex of the stem mostly resemble those of *E. laevigatum*. This pattern and its relevance to our machine learning approach are discussed in later sections.

Our classification system mimics the human approach described above by splitting the computational pipeline into two stages. The first stage *detects* aerial stem nodes and strobili in the image under analysis, and a simple computation collects statistics of these detection results. This stage also learns to categorize a node into either a hyemale‐type (H) node or a laevigatum‐type (L) node, learning this distinction exclusively from images of *E*. *hyemale* and *E. laevigatum* specimens that were expertly annotated by a human. The second stage computes simple statistics of node occurrences, describing the 10 nodes in an image that were detected with the highest confidence. This stage then takes these statistics as input and *classifies* the entire image into one of the three taxa.

Although we annotated both nodes and strobili for this study, in practice, we only detected nodes in our experiments, because an image typically has many nodes but few strobili. The paucity of strobili in our preliminary data set made it impossible to train a detector, given the small number of annotated images we had at our disposal (Table 1). We hope to reintroduce strobili once we gain access to more annotations in future work. We describe detector, detection statistics, and classifier below.

### Detection network architecture

We used the Single Shot Multibox Detector (SSD) with a VGG16 base classification network (Liu et al., 2016) to simultaneously detect and classify stem nodes as hyemale‐type (H) nodes or laevigatum‐type (L) nodes. Older detectors would first hypothesize where an object (a node in our case) might occur, and then classify it into one of the possible categories (H or L in our case). In contrast, the SSD merges localization and categorization into a single step. Specifically, it superimposes a grid of regular sample points on the image. The pitch of the grid is equal to the size of the smallest object one expects to find (30 pixels in our case). For each point on the grid, the detector examines rectangles centered at the grid point and of different sizes and aspect ratios. We used six sizes and six aspect ratios, for a total of 36 boxes per grid point. The SSD then classifies each box into H or L and computes a *score* for this classification. The score measures the network’s confidence in the classification result. Boxes that are well centered on an actual node are expected to have a high score, and other boxes are not. Each high‐score box is output as a detection, together with the category (H node or L node) that yielded the maximum score for that box. A box is not output if it has a score that, although high, is lower than that of another box with which it overlaps. This criterion prevents overlapping boxes to be output. By combining detection and classification into a single neural network, the SSD has been shown to be easier to train and more accurate than systems that separate the two tasks from each other (Liu et al., 2016).

### Detection statistics

The node detector was trained exclusively on confirmed *E. hyemale* and *E. laevigatum* specimens, in which human annotators drew boxes around all or most stem nodes in 108 images. Any node that appears in an *E. hyemale* image is automatically labeled as an H node. Similarly, any node that appears in an *E. laevigatum* image is automatically labeled as an L node. During testing, on the other hand, the detector analyzed images of any of the three taxa, including the hybrid *E. ×ferrissii*. The idea is that most nodes in an *E. hyemale* image will be classified as H nodes, most of those in an *E. laevigatum* image as L nodes, and the nodes in *E. ×ferrissii* images will be a mixture of the two types. As we show in the section on results below, data largely support this conjecture.

To capture useful information about the distribution of node types (H or L) in an image, the detector does not just return a box and a label, but also a confidence score. This score is technically a real number between 0 and 1. However, to make scores more legible in Fig. 2, we multiplied all scores by 10 and rounded them to the nearest integer, so that a score is now an integer between 0 (no confidence) and 10 (full confidence). For instance, a score of 0.73 would be displayed as 7.

Given this scoring system, for each image, we selected 10 top‐scoring H nodes, as well as 10 top‐scoring L nodes, and computed the average score in each of these two sets. These two numbers, a_L_ and a_H_, are the information we provided to the image classifier, as explained below. We also experimented with different combinations of statistics, such as the ratio of the two average scores or the two standard deviations. However, the two averages proved empirically to work best.

### Classification

It is currently impossible for us to train a deep network for classification because of the small number of manually annotated images at our disposal. Instead, we experimented with a number of simpler classifiers: a logistic‐regression classifier, a decision tree, and a *k*‐nearest‐neighbor classifier.

A logistic‐regression classifier finds straight‐line boundaries in the plane defined by the two averages a_L_ and a_H_ that best separate the three classes (*E. hyemale*, *E. laevigatum*, *E. ×ferrissii*). A decision tree classifier takes the two values a_L_ and a_H_ and asks questions of the form “is a_L_ (or a_H_) greater or smaller than threshold t”, where t is one of several values generated in the process of training the decision tree. The classifier then returns one of three classes (*E. hyemale*, *E. laevigatum*, *E*. ×*ferrissii*) depending on the answers to these questions. Finally, a *k*‐nearest‐neighbor classifier simply memorizes the pair (a_L_, a_H_) for each of the training images. Given a new test image with pair (a′_L_, a′_H_), the classifier then assigns to it the majority class among the *k* training images whose values of (a_L_, a_H_) have smallest distances from (a′_L_, a′_H_). We found decision trees and 5‐nearest‐neighbor classifiers to work best in our experiments.

### Training

As is customary, we used three annotated sets of data. The *training set* is the group of manually annotated images that are used for training. The *validation set* is a separate group of manually annotated images that are used to decide when the training algorithm is to stop; as training progresses, the current classifier is repeatedly evaluated on the validation set, and training stops when this evaluation returns optimal results. Finally, the *test set* is a third group of manually annotated images that are used to evaluate the performance of the classifier. It would be misleading to report error rates on the validation set as the classifier’s performance, because the training algorithm had a chance to “peek” at the validation data. The training set, on the other hand, is pristine, and performance on it is a less‐biased estimate of how well the classifier analyzes previously unseen data.

We annotated 108 images, as described earlier, 36 for each taxon. We reserved half the images for training, 24 for validation, and 30 for testing. Figure 2 shows a few sample annotations from the test set. We trained the detector using the mini‐batch gradient descent method with a batch size of eight images and a fixed learning rate of 0.0001 (Bottou, 1998). We used a technique called *data augmentation* to provide greater data variety to the training method (Krizhevsky et al., 2012). Specifically, each specimen image used in training is rotated by a random angle, and a random 500 × 500‐pixel sub‐image is cropped from it and fed to the training algorithm. The bounding‐box annotations are appropriately rotated and translated to match each rotated and cropped image. If this procedure is repeated multiple times for each image, it effectively creates new (albeit artificial) examples and augments the training set as a result. This method is routinely used in machine learning and training converged in about 2000 iterations in our experiments. As is usually done in deep learning (Goodfellow et al., 2016), convergence is declared when an error rate computed on the validation set starts to increase.

### Software

We wrote our code in Python and used the PyTorch machine learning framework (Paszke et al., 2019). The source code and data are available at https://github.com/Xiaohan‐Wang/Equisetum_classification_3.

## RESULTS

Here we describe the performance of the node detector first, and then that of the classifier. As part of our discussion, we also test our conjecture that some *E*. *×ferrissii* nodes look like *E. hyemale* nodes and others look like *E. laevigatum* nodes, rather than having their own separate appearance specific to *E*. *×ferrissii*.

### Node detection

The detector can be wrong in several ways: (1) false positives: nodes are detected where none exist; (2) false negatives: the detector fails to report an existing node; (3) nodes in *E. hyemale* images are reported as L nodes; and (4) nodes in *E. laevigatum* images are reported as H nodes. Reported labels for nodes in *E. ×ferrissii* images, on the other hand, are never inherently right or wrong, as they are neither *E. hyemale* nor *E. laevigatum* nodes per se. Figure 2 gives anecdotal examples of errors of the first two types. As we show below, errors of the last two types (3 and 4) are extremely rare. We do not measure the rate of false negatives, as this would require that all images be fully annotated. The example on the right in Fig. 2 illustrates vividly that this is not uniformly the case.

Errors 3 and 4, that is, cross‐type misclassifications, are much more important, because the classification of images into species depends on a reliable distinction between H nodes and L nodes. Table 2 below shows the number of true and detected nodes for *E. hyemale* and *E. laevigatum* test images, which are the only images for which a “true” node type can be defined. Of the 272 true nodes detected in these 30 test images, only six are misclassified, resulting in a 98% cross‐type accuracy.

**Table 2:** The number of true and detected nodes of type H (hyemale) and L (laevigatum) in *Equisetum hyemale* and *E. laevigatum* test images. Of the 272 true nodes detected in 30 test images, only six are misclassified, resulting in a 98% cross‐type accuracy.

		Detected	
		*E. hyemale* image	*E. laevigatum* image
**True**	H node	145	4
	**L node**	2	121

To test our conjecture that the majority of *E*. *×ferrissii* nodes do not have their own specific appearance, we re‐trained our detector with three classes of nodes instead: H and L, as before, plus F nodes to denote nodes that appear in *E*. *×ferrissii* images. Just as for the other two species, we reserved 18 *E*. *×ferrissii* images for training and 10 for testing. Other than this difference, the training procedure was the same as above.

Table 3 shows the confusion matrix for the three‐class experiment, where it can be inferred that H nodes and F nodes are often confused with each other (36 times out of 229), while L nodes are quite distinctive (six errors out of 96 cases). The cross‐type accuracy is now 87%, significantly lower than the 98% rate for the two‐species experiment.

**Table 3:** Confusion matrix analogous to that shown in Table 2 when *Equisetum ×ferrissii* nodes are considered a class of their own. Nodes in *E. laevigatum* images are classified incorrectly only six times out of 96, while nodes of the hybrid species *E*. *×ferrissii* and of its parent species *E. hyemale* are confused with each other in 36 out of 229 cases.

		Detected		
		*E. hyemale* image	*E. laevigatum* image	*E*. *×ferrissii* image
**True**	**H node**	85	5	22
	**L node**	0	90	0
	**F node**	14	1	108

The confusion between *E*. *×ferrissii* and *E. hyemale* nodes in this experiment suggests that working with two node types rather than three may be more reliable. On the other hand, the difference in cross‐type accuracy reported above is not by itself conclusive evidence that introducing F nodes is useless. Because of this, we also perform image classification experiments in both scenarios (two node types or three node types) as described next.

### Image classification

Table 4 shows the confusion matrix for the image classification results based on two node types (H and L, see Table 2), using a 5‐nearest‐neighbor classifier. *Equisetum hyemale* images are correctly classified in nine out of 10 cases, *E. ×ferrissii* images are correctly classified in eight out of 10 cases, and *E. laevigatum* images are never confused. The resulting accuracy is 90%.

**Table 4:** True image classes (rows) versus classes predicted by a 5‐nearest‐neighbor classifier (columns) based on the average scores a_H_ and a_L_ for the top 10 H nodes and the top 10 L nodes detected in each test image. Out of 30 test images, 27 are classified correctly, for a classification accuracy of 90%.

		Predicted		
		*E. hyemale*	*E. laevigatum*	*E*. *×ferrissii*
**True**	***E. hyemale***	9	0	1
	***E. laevigatum***	0	10	0
	***E*. *×ferrissii***	2	0	8

As discussed in the section on node detection above, using three node types (H, L, F) as opposed to just two (H, L) is likely to be generally unreliable, because of the confusion between H and F node types documented in Table 3. However, one cannot conclude based on that experiment alone that using three node types would lead to a worse outcome. Because of this, we also trained a logistic‐regression classifier, a decision tree, and *k*‐nearest‐neighbor classifiers for several values of *k* using scores a_H_, a_L_, a_F_ for the top 10 nodes of type H, L, and F detected in each image, similarly to what we did for the experiment in Table 3.

In this experiment, the decision tree classifier ended up winning by a small margin over the best *k*‐nearest‐neighbor classifier, which still turned out to use five neighbors. Classification results for the decision tree classifier when three node types are used are shown in Table 5.

**Table 5:** True image classes versus classes predicted by a decision tree based on the average scores a_H_, a_L_, and a_F_ for the top 10 H nodes, top 10 L nodes, and top 10 F nodes detected in each test image. Out of 30 test images, 27 are classified correctly, for a classification accuracy of 90%. This accuracy is the same as for the two‐node experiment in Table 4.

		Predicted		
		*E. hyemale*	*E. laevigatum*	*E*. *×ferrissii*
**True**	***E. hyemale***	8	1	1
	***E. laevigatum***	0	10	0
	***E. ×ferrissii***	1	0	9

The classification accuracy is still 90%, which is the same as that obtained using only H and L nodes (Table 4). It is, of course, difficult to extrapolate results on this small number of images to experiments on a larger scale. Nonetheless, evidence that *E. hyemale* nodes are easily confused (15.7% of the time) with some *E*. *×ferrissii* nodes is fairly clear from Table 3.

## DISCUSSION

We built computational models to detect phenotypic structures (aerial stem nodes) that are relevant to *Equisetum* identification to assess whether we could potentially automate and enhance the identification of three closely related taxa that are commonly misidentified in herbaria. Within these models, node detection works well, with 98% accuracy across L nodes and H nodes in *E. laevigatum* and *E. hyemale* images (Table 2).

Our experiments only partially support the conjecture that some *E*. *×ferrissii* nodes look like *E. hyemale* nodes and others look like *E. laevigatum* nodes. Specifically, image classification experiments based either on two node types or three give the same 90% accuracy (Tables 4, 5). However, our detection experiments show quite clearly (Table 3) that *E*. *hyemale* and *E*. *×ferrissii* nodes are confused quite often (15.7% of the time). This suggests that working with only two node types rather than three may lead to more reliable results.

Several avenues of investigation present themselves to further improve on our results. It is likely that better results will be achieved by a more nuanced way to capture statistics of node distribution. Specifically, we plan to develop ways to concatenate the actual node patches from an input image into a detailed description of all the nodes and feed this rich description to a neural net for classification. Training this net will require reliable image‐level labels, that is, an authoritative classification of each image into one of the three taxa, preferably using strobilus characters for corroboration.

Eventually, we plan to investigate to what extent information about the relative *location* of each detected node along the stem contributes to improving classification results. To determine this location, we plan to write code that approximates assessing the stem node information in the same way that an expert does when making an identification. The code will (i) segment the plant from its background; (ii) separate different stems from one another, even when they intersect or overlap in the image; (iii) extract a skeleton of the shape of each stem; and (iv) encode the relative position of each node along the skeleton. Step (i) may benefit from recent specimen segmentation methods (White et al., 2020). Based on our own personal observations, we estimate that this additional information will much improve identification because some number of H nodes tend to appear toward the base of the stem in *E. ×ferrissii*, and L nodes tend to be more predominant distally. This can be clearly observed in the center image of Fig. 2, which exhibits red (H) boxes toward the stem base, and blue (L) boxes toward the top.

Moving forward, we expect that strobili will also be informative, but we had to ignore them in this first implementation because they are much less numerous than stem nodes in specimen images and so did not provide sufficient training data. In sum, data matters. Machine learning involves the study and construction of computer algorithms that can learn and make predictions based on data. Enough data will allow us to use detection and classification models with a large number of parameters (such as neural networks), and accurate results will be, in large part, a function of how much data we are able to manually annotate prior to applying computer vision approaches.

A recent paper (Wäldchen and Mäder, 2018) surveys 77 conference articles and 43 journal articles published between 2005 and 2015 on plant species identification using computer vision techniques. Of these 120 papers, 106 use exclusively leaves for discrimination between species, 13 use only flowers, and a single article (Joly et al., 2014) considers various parts (flowers, leaves, fruit, bark, and a full view of each plant) simultaneously. These 120 studies distinguish among a number of species ranging from two or three to several hundred. One notable exception is Wu et al. (2015), which classifies up to 23,025 different species based on leaf morphology and evaluates the system on more than one million images, achieving approximately 92% accuracy.

Our contribution is obviously not in competition with these methods. Instead, we focused on a specific problem in *Equisetum* where botanists struggle to identify taxa reliably, and we investigated stem nodes as the most useful part of the plant for classification of *E*. *hyemale*, *E. laevigatum*, and *E*. *×ferrissii* specimens. None of the studies mentioned above includes *Equisetum*, and it is unlikely that leaf shape by itself would be useful to address the problem we studied. We did not aim to build a system that scales to thousands of species and millions of images. Instead, we showed an example of the type of methods that may have to be brought to bear on the problem of distinguishing among subtly different species, once ≥90% of the distinctions are made correctly by methods like the ones cited above. Our goal was to capture, in a computer algorithm, the type of specialized expertise that might help solve some of the more subtle identification problems shared by many botanists.

Making herbarium specimens accessible online as digital data and images is in itself a tremendous achievement. However, novel uses of these data ultimately rely on the specimens being properly identified. Our aim in pursuing this exploratory exercise, which combines advances in deep learning with digitized herbarium images, was to investigate whether it was feasible to develop an automated approach to accurately identify species that are often misidentified. There are too few botanical experts and too many improperly identified specimens to ever hope to resolve this conundrum using traditional methods (Drew, 2011; Deng, 2015; Goodwin et al., 2015). However, we are encouraged here by our preliminary results on using computer vision to distinguish among *E. hyemale*, *E. laevigatum*, and *E. ×ferrissii––*a common and widespread hybrid between them. We are also motivated to pursue more nuanced machine learning approaches that will focus computer models on the same phenotypic elements that botanical experts find most informative for species classification.

## AUTHOR CONTRIBUTIONS

K.M.P. and M.D.W. performed manual annotations on digitized images of *Equisetum*. K.M.P., M.D.W., C.T., E.K.M., and X.W. designed the study, performed analyses, and wrote the paper. C.T. and X.W. designed and implemented machine learning approaches.

## Supporting information


**APPENDIX S1.** Links to herbarium specimen images and metadata downloaded from SERNEC (http://sernecportal.org/portal/) for each of the three focal taxa (*E. hyemale*, *E. laevigatum*, and *E. *×*ferrissii*).

## Data Availability

The source code and data are available at https://github.com/Xiaohan‐Wang/Equisetum_classification_3. Links to herbarium specimen images and metadata downloaded from SERNEC (http://sernecportal.org/portal/) for each of the three focal taxa (*Equisetum hyemale*, *E. laevigatum*, and* E. *×*ferrissii*) are published in Appendix S1, and are also available at https://github.com/Xiaohan‐Wang/Equisetum_classification_3.
